# USE OF MOTIVATIONAL INTERVIEWING TO INTERVENE ELDER ABUSE: A RANDOMIZED CONTROL TRIAL

**DOI:** 10.1093/geroni/igae098.0723

**Published:** 2024-12-31

**Authors:** Elsie Yan, Louis To, Debby Wan, Jasmin He, Daniel W L Lai, David Burnes, Karl Pillemer, Mark Lachs

**Affiliations:** The Hong Kong Polytechnic University, Hong Kong, Hong Kong, China (People’s Republic); The Hong Kong Polytechnic University, Hong Kong, Hong Kong, China (People’s Republic); The Hong Kong Polytechnic University, Hong Kong, Hong Kong, China (People’s Republic); The Hong Kong Polytechnic University, Hong Kong, Hong Kong, China (People’s Republic); Hong Kong Baptist University, Hong Kong, Hong Kong; University of Toronto, Toronto, Ontario, Canada; Cornell University, Ithaca, New York, United States; Weill Cornell Medicine, New York, New York, United States

## Abstract

Elder abuse causes serious harm to individuals, families, and society. There is an urgent need to develop and test appropriate interventions to tackle it. This study tested the effectiveness of a motivational interviewing (MI)-based intervention in reducing elder abuse severity and promoting positive changes among community dwelling older Chinese in Hong Kong. The intervention involved a 90-minute individual engagement session and three 30-minute booster sessions delivered by trained MI facilitators. Personal goals for improvement of known risk factors such as poor physical and psychological health, lack of social support were set out followed by the application of MI processes. Pre- and post-test comparison of 33 intervention cases and 28 control cases are presented here. Analysis of pre-treatment differences found significant difference between the two groups in their monthly income, number of offspring and co-residing persons, physical frailty, general self-efficacy and abuse severity. Difference in differences technique was applied to mitigate the effects of extraneous factors and selection bias. Results show that intervention group performed significantly better than the control group upon completing the intervention program. Significant between group differences were found in terms of changes in psychological distress (GHQ, z=-3.043, p< 0.05), general self-efficacy (GSES, z=-4.988, p< 0.05), social support (MSPSS, z=-3.080, p< 0.05), abuse severity (CTS2, z=-2.593, p< 0.05), and perceived ability to change (z=-3.749, p< 0.05). MI has demonstrated good potential for elder abuse intervention. Further study should be conducted to identify key elements in MI leading to positive changes and refine the model for elder abuse prevention and intervention.

